# Introduction of CTA-index as Simplified Measuring Method for Thrombus Perviousness

**DOI:** 10.1007/s00062-020-00957-4

**Published:** 2020-09-29

**Authors:** Maria Berndt, Fabian Mück, Christian Maegerlein, Silke Wunderlich, Claus Zimmer, Stefan Wirth, Sebastian Mönch, Johannes Kaesmacher, Benjamin Friedrich, Tobias Boeckh-Behrens

**Affiliations:** 1 Department of Diagnostic and Interventional Neuroradiology, Klinikum rechts der Isar, School of Medicine, Technical University of Munich, Munich, Germany; 2 Department of Neurology, Klinikum rechts der Isar, School of Medicine, Technical University of Munich, Munich, Germany; 3 Department of Radiology, Helios Klinikum München West, Munich, Germany; 4 Clinic and Polyclinic for Radiology, Ludwig-Maximilians-University of Munich, Munich, Germany; 5 Department of Radiology and Nuclear Medicine, Schwarzwald-Baar Klinikum, Villingen-Schwenningen, Germany; 6 Department of Neuroradiology, Inselspital, University Hospital Bern, University Bern, Bern, Switzerland

**Keywords:** Stroke, CT-angiography, Thrombus characteristics, Perviousness

## Abstract

**Purpose:**

Thrombus features on admission CT are useful imaging markers for clot characterization, stroke pathogenesis and outcome prediction. In this context, thrombus perviousness is a promising parameter, but reliable assessment in daily clinical practice is demanding. The aim of the present study was to evaluate an easy to assess measuring method for thrombus permeability at the time of admission.

**Methods:**

The CTA-index, which measures relative thrombus attenuation on admission CTA, was compared to the known perviousness parameter in a cohort of 101 patients with large-vessel occlusions of the middle cerebral artery and correlated to clinical outcome parameters (mRS after 90 days, ≤2 rated as favorable). For validation, this correlation was tested in a second independent cohort (*n* = 87), and possible associations between the CTA-index and outcome measurements (NIHSS/mRS/mTICI) were assessed.

**Results:**

In the first cohort a coherence between conventional perviousness measurements and the CTA-index was shown. The CTA-index differed significantly between favorable (−0.55 ± 0.16) and non-favorable outcomes (−0.64 ± 0.14, *p* = 0.01). In the validation cohort this result could be independently reproduced (−0.52 ± 0.13/−0.70 ± 0.09, *p* < 0.01). The CTA-index showed an association with low NIHSS at discharge (*p* < 0.01), favorable outcome after 90 days (*p* < 0.001) and with better reperfusion (measured by mTICI score, *p* = 0.04).

**Conclusion:**

The CTA-index is an easy to assess imaging parameter on admission CTA in the acute stroke phase and is associated with angiographic and clinical outcome. It can be considered as a simplified measuring method for thrombus perviousness, which is known to provide useful information for further stroke progress and clinical course as well as therapeutic and rehabilitative decisions.

## Introduction

Intracranial large vessel occlusions (LVO) cause more than one third of acutely presenting ischemic strokes but lead to disproportionately high stroke-related mortality, severe impairments, disability and consequently poor quality of life [1, 2]. Recent therapeutic developments aimed for immediate and complete reperfusion of an acute LVO on the basis of the established treatment of endovascular mechanical thrombectomy as the current standard procedure [3–7]. Recently, thrombectomy has been proven to be effective also in later time windows and smaller vessels [8–10]. For an optimal adaption of therapeutic and rehabilitative processes, meaningful imaging parameters in the early stroke phase before initiation of treatment might be useful.

On admission CT imaging was used in previous studies to assess thrombus characteristics, such as length, density, perviousness and even radiomic features [11–17]. These features were demonstrated to be useful imaging markers for clot characterization, stroke pathogenesis and outcome prediction, while a particular focus was set on thrombus perviousness [14, 18–20]; however, accurate perviousness measurement in its current form needs additional co-registration processes in the acute image analysis, which are not widely available. Previous studies about clot characteristics supported the usefulness of an automatic assessment of thrombus characteristics [21]. Thus, comfortable imaging parameters are needed that are applicable without the necessity of not fully workflow-integrated software.

The aim of the present study was to evaluate a parameter that can easily be assessed and utilized by the primary radiologists in the acute setting of an LVO. To address this, the new parameter CTA-index is introduced, intended as surrogate marker for thrombus permeability. This parameter measures the relative thrombus attenuation on an admission CTA without the need to co-register different CT scans as is required for the previously described perviousness assessment [18].

After comparing CTA-index to conventional perviousness parameters with the intention to use it as a simplified measurement, the CTA-index was tested as a possible independent outcome predictor and its equivalency to the already established perviousness parameter was examined. In a second, independent cohort, interrater agreement for the assessment of CTA-index was tested, and the associations between CTA-index and angiographic as well as clinical outcome were examined.

## Material and Methods

As primary end point correlations between already gathered thrombus perviousness measurements (change in thrombus attenuation/void fraction) and the newly introduced CTA-index were tested in the first group of patients with an acute LVO of the MCA, labeled as the perviousness cohort. The impact of the CTA-index was further evaluated by comparing its values between patients with favorable and non-favorable clinical outcome. For validation purposes, the association of the CTA-index with angiographic and clinical outcome parameters was tested in a second independent group of patients with an acute LVO of the MCA, labeled as validation cohort.

The local ethics committee gave the project a positive vote under number 250/17 S and the need for patient consent was waived.

### Sample and Patient Description

The perviousness cohort (*n* = 101) consisted of a homogeneous collective of patients with an acute LVO of MCA, who were consecutively treated at a single comprehensive stroke center between January 2015 and December 2017 after applying specified exclusion criteria as described in a previous work [18]. The independent validation cohort (*n* = 87) consisted of an analogous collective of patients with an acute LVO of MCA, who were consecutively treated at the same single comprehensive stroke center between January and December 2018 (total number *n* = 158). The following exclusion criteria were applied: MCA occlusion beyond the proximal M2 segment, that makes permeability assessment impossible due to small vessel lumen (*n* = 4 excluded), incomplete occlusion with proof of residual blood flow (*n* = 5 excluded), missing or insufficient preinterventional imaging (CTA) with a thickness >3 mm (*n* = 62 excluded); data were evaluated by a neuroradiologist with at least 3 years of experience (MB, blinded).

Basic demographic, clinical, and interventional data of patients were gathered and are summarized in Table 1. Certified neurologists assessed the National Institutes of Health Stroke Scale (NIHSS) score at the time of admission (pretreatment) and discharge (posttreatment). The modified Rankin scale (mRS) was used to measure disability at day 90. Favorable outcome was defined as mRS ≤2, and non-favorable as mRS >2.

**Table 1:** Baseline demographic, clinical and interventional data for the perviousness and validation cohort

Characteristics	Perviousness cohort (*n* = 101)	Validation cohort (*n* = 87)
*Age, years, median (IQR)*	78 (71–86)	77 (69–84)
*Sex, n (female/male)*	49/52	53/34
*mTICI score postrecanalization (n)*		
0	12	6
1	1	2
2a	5	2
2b	33	25
3	50	52
*Procedure time (min, median/IQR)*	35 (22–49.5)	31 (21–62)
*Additional intravenous thrombolysis (n (%))*	48 (47.5%)	31 (35.6%)
*NIHSS (median, IQR)*		
Pretreatment	14 (10–18)	13 (7–18)
Posttreatment	6 (1–12)	5 (1–13)
*mRS score (90 days), n*	*n* = 51	*n* = 81
0–2	23	30
>2	28	51

*IQR* interquartile range, *NIHSS* National Institutes of Health Stroke scale, *mTICI* modified thrombolysis in cerebral infarction, *mRS* modified Rankin Scale

The modified thrombolysis in cerebral infarction (mTICI) score [22] was determined by two experienced neurointerventionalists (CM, BF) in consensus. Successful recanalization was defined as mTICI 2b–3. Time of the groin puncture, time of reperfusion and corresponding procedure times were taken from the existing database. Procedure time was defined as difference between the time of groin puncture and reperfusion. In cases where recanalization was not successful (mTICI <2b), the control series after the last maneuver was used as the time endpoint.

### Admission Imaging Technique

For all included patients in both cohorts, native CT and CTA imaging were performed before mechanical recanalization. For patients who were examined in our hospital (90%/60% within the perviousness/validation cohort), standard non-helical cerebral noncontrast computed tomography (nCT) was performed either on a 64-row or 256-row CT scanner (Philips Brilliance 64/Brilliance iCT; both Philips Medical Systems B.V., Best, The Netherlands) by use of 120 kV, 170 mAs, with 2.5-mm section thickness. The CTA was performed as a helical scan, with coverage from vertex to the aortic arch and continuous axial sections parallel to the orbitomeatal line with 0.6 mm section thickness. A dual head power injector (Medrad, Warrendale, PA, USA) with an 18‑G i.v. access was used for contrast medium injection. A bolus of 70 mL Imeron 400 (Bracco Imaging Deutschland GmbH) at a flow rate of 4 mL/s was applied, followed by 50 mL NaCl. The scan was autotriggered by the appearance of contrast in a region of interest manually placed in the ascending aorta (attenuation >150 HU).

### Thrombus Perviousness and CTA-index Assessment

**Fig. 1:**
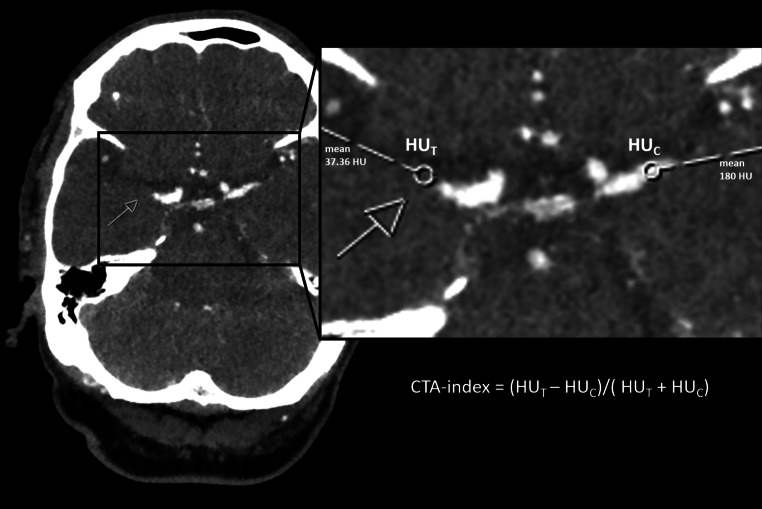
Example of CTA-index assessment for a patient that underwent admission CTA imaging of an acute occlusion of the right middle cerebral artery within the M1 segment. The ROIs for measurements of mean HU were placed 1.5 mm behind the occlusion site (T) and at a corresponding position of the contralateral, not occluded vessel (C)

Within the perviousness cohort, the thrombus perviousness parameter void fraction (ε = ∆t/∆c) was assessed by calculating the ratio of change in thrombus attenuation within the thrombus (∆t = HU_CTA_-HU_CT_) and in the contralateral artery (∆c). Change in thrombus attenuation was measured by the increase of Hounsfield units (HUs) between the native CT and CTA scans after automatic alignment using a rigid registration method (for further details see [18]). In the same patient cohort, the CTA-index was assessed by measuring relative thrombus attenuation on admission CTA. The CTA-index was also assessed in the validation cohort by a neuroradiologist with 3 years experience (MB) and blinded to all clinical data. In consideration of appositional thrombus growth, measuring points were placed 1.5 mm distal of the visible stop of contrast agent (occlusion site T) and at a corresponding position of the contralateral, not occluded vessel (C) as was previously described for perviousness measurements [18]. Mean HUs of the circular ROIs were extracted and relative thrombus attenuation was calculated using the following formula: CTA-index = (HU_T_ − HU_C_/(HU_T_ + HU_C_), see Fig. 1. This kind of asymmetry index was previously used in stroke studies, particularly in structural imaging studies, to correct for interindividual differences and it appeared as a popular predictor variable [23–25].To test for inter-observer agreement, a senior radiologist with six years of experience (FM), blinded to all clinical data and the analysis performed, repeated the measurements of the CTA-index in the validation cohort.

### Statistical Analysis

Wilcoxon rank-sum tests were used for comparison of the CTA-index between patient groups with favorable/non-favorable outcome at day 90. After performing a linear regression to get residuals of the CTA-index (controlling for age, sex, CTA-image slice thickness, recanalization success measured by mTICI and procedure time), a Spearman rank bivariate correlation was performed between residuals of the CTA-index and NIHSS at discharge. A binary logistic regression was performed to test the association of favorable outcome at day 90 with the CTA-index, adjusting for age, sex, CTA-image slice thickness, recanalization success measured by mTICI and procedure time. A partial correlation analysis was performed to test a possible association between the CTA-index and mTICI score, controlling for age and slice thickness.

To evaluate interrater agreement, an intraclass coefficient (ICC) analysis was performed between the measurements of the CTA-index, gathered independently by a neuroradiologist with 3 years of experience (MB) as well as a radiologist with 6 years of experience (FM).

All statistical analyses were performed using IBM SPSS Statistics (version 25, IBM Corp, Armonk, NY, USA).

## Results

### Patient Characteristics

In total, 188 patients (101 in the perviousness cohort, 87 in the validation cohort) met the required inclusion criteria. An overview of demographic, clinical and interventional data of patients can be found in Table 1.

### CTA-index in Comparison to Conventional Measurements of Perviousness

**Fig. 2:**
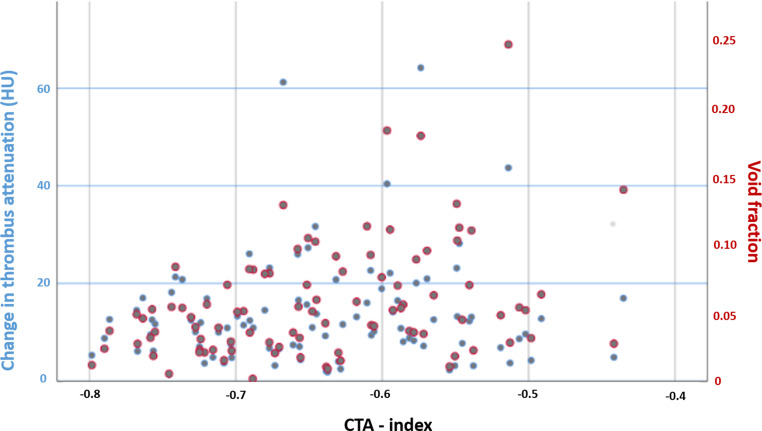
Association between CTA-index values and the conventional perviousness measures. Change in thrombus attenuation (*blue*) and void fraction (*red*)

Fig. 2 shows the associations between perviousness measurements (change in thrombus attenuation and void fraction) and CTA-index in the perviousness cohort.

**Fig. 3:**
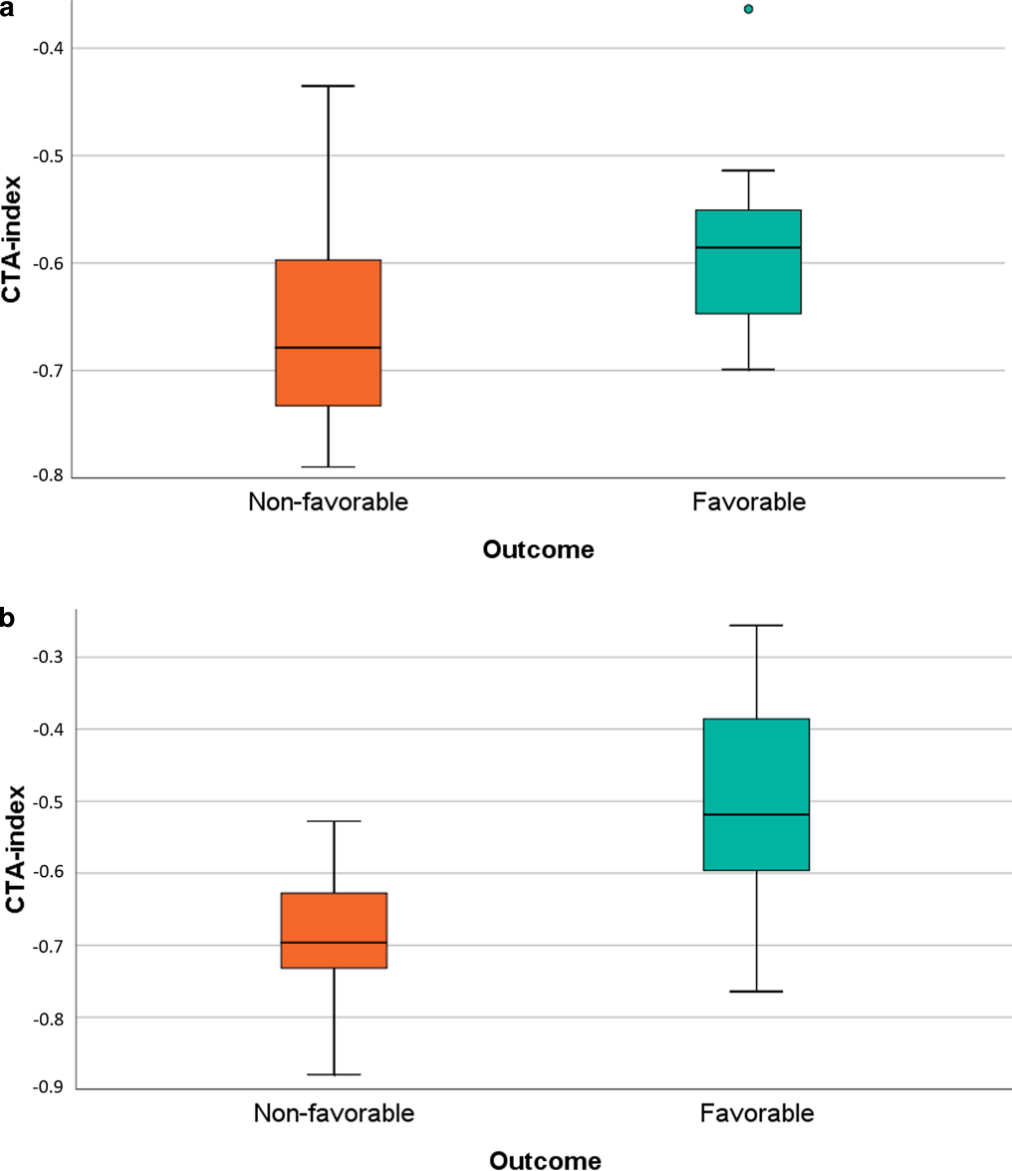
Distribution of CTA-index for different outcome groups (measured by mRS at 90 days, favorable = mRS ≤2, non-favorable = mRS >2) for the perviousness cohort (**a**) and validation cohort (**b**)

Significantly higher CTA-index values were found for patients with favorable (−0.55 ± 0.16) than non-favorable outcome (−0.64 ± 0.14, *p* = 0.01), see Fig. 3a).

### Validation of CTA-index as a Variable Associated with Clinical and Angiographic Outcome

Comparisons of the CTA-index between different outcome groups were repeated within the independent validation cohort. Compared to the perviousness cohort, corresponding values of the CTA-index were found for favorable/non-favorable outcome after 90 days (−0.52 ± 0.13 /−0.70 ± 0.09, respectively), with significantly higher values for favorable outcome (*p* < 0.01), see Fig. 3b.

In a logistic regression model, favorable outcome after 90 days was significantly associated with the CTA-index (R^2^ 0.64, *p* < 0.001), while other variables such as age, sex, CTA-image slice thickness, recanalization success (measured by mTICI) and procedural time showed no significant impact on the analysis.

In a correlation analysis, residuals of the CTA-index (corrected for all covariates mentioned earlier) were negatively correlated to NIHSS at discharge (r = −0.3, *p* = 0.007).

In a separate analysis, a higher CTA-index was correlated to higher mTICI score (r = 0.22, *p* = 0.04).

### Reliability of CTA-index Assessment

In the analysis of interrater reliability for the measurements of the CTA-index, a good interrater agreement was observed (ICC of 0.87, confidence interval CI 0.81–0.91). This suggests a good reliability of the CTA-index measurement within the study setting with observer independency.

## Discussion

The present study introduces a new imaging marker, which is easily and quickly achievable using the admission CT imaging of an acute LVO. The CTA-index measures the relative thrombus attenuation in CTA imaging and proved to be usable as a simplified parameter of perviousness. While the impact of established but more complex perviousness measurements on clot characterization, stroke pathogenesis and especially clinical outcome is well-known, the CTA-index proved to have the capacity of outcome prediction as well. In a second independent cohort, a trend to better angiographic and clinical outcome was shown for cases of higher CTA-index. The CTA-index can easily be assessed in the acute stage of an ischemic stroke within the clinical workflow at the time of admission imaging and might replace perviousness measurements with respect to its outcome prediction capacity.

For validation purposes, the clinical workflow of the admission CTA acquisition of an acute stroke was simulated: A neuroradiologist with 3 years of experience and a radiologist with 6 years of experience performed the measurements independently on computers for radiological assessment. It was shown that both radiologists, who are involved in diagnosing acute LVOs, are able to assess the CTA-index as a simplified thrombus perviousness measure. The high interrater reliability underlines the possible applicability in daily clinical practice, independently from specialized neuroradiological expertise.

The prerequisite measurements for the new parameter are standardly collected in arterial-phase CTA because of its wide availability and general use in the acute setting of an LVO. Regarding perviousness measurements, different opinions about optimal techniques exist. While one study [19] favored the dynamic CTA for perviousness assessment due to better outcome prediction after reperfusion therapy than conventional single-phase CTA, another study [26] showed that the use of an arterial-phase CTA appeared to be most suitable for the measurement of thrombus perviousness compared to delayed phases. Arterial-phase CTA is broadly available and commonly used in stroke centers, an essential requirement for a parameter that is intended to be used within the clinical workflow.

If effortful acquisition or image postprocessing is necessary, a broad acceptance in clinical practice seems unlikely. Therefore, CTA-index has one key advantage over perviousness measures: it does not require a co-registration process [14, 18, 26]. It is based on a standard imaging technique of the clinical routine. A previous study showed a practical method by omitting the co-registration process [20]. Visual co-registration carries the risk of incorrect measurements in native CT scan due to artifacts or imprecise identification of the sometimes hard to identify clot. The measurement of CTA-index does not need native CT scans and is therefore independent of the image quality of native CT scans.

The findings in the present work are perfectly in line with a previously published study that compared different methods of thrombus permeability measurements and demonstrated superiority of a simplified measure of perviousness solely based on CTA imaging [27]. The study showed that such simple methods for assessing thrombus perviousness are as reliable as more advanced image processing methods and can help physicians reasonably discriminate between “recanalizers” and “non-recanalizers” after iv-lysis [27]; however, that study did not analyze if thrombus perviousness may influence recanalization success of mechanical thrombectomy, a question we could further clarify with the present study: The CTA-index is associated with the recanalization success of thrombectomy, showing that higher relative thrombus attenuation in CTA imaging was associated with higher mTICI scores, and additionally with improved clinical outcome parameters. Both NIHSS at discharge as well as the long-term outcome after 90 days were associated with the CTA-index, consistent with previous studies using perviousness assessment in conventional single-phase CTA [14, 28].

In the interpretation of our results, it is necessary to keep in mind that these are purely observational findings, possibly associated with different pathophysiological backgrounds. Unlike perviousness, measuring thrombus permeability by assessing the attenuation increase between native and angiographic CT images, the CTA-index measures the relative thrombus attenuation without subtraction of thrombus density values in native CT scans. That means that the native thrombus density also affects the CTA-index. The CTA-index likely combines two different elements that are separately and probably independently associated with the same clinically important parameters “recanalization success” and “outcome”: despite controversies in the literature, some studies showed an association of higher thrombus intensities with good angiographic outcomes [29], and also higher perviousness was associated with improved outcomes [14, 19]. Both findings are probably based on pathophysiologically different mechanisms. The CTA-index assessment combines these associations and thereby may increase the power for pointing out an association with recanalization success and outcome.

In conclusion, we are convinced that the usage of this easily assessable parameter might be of high clinical benefit, as the CTA-index is simple to acquire for radiologists during the standard workflow before any therapeutic decisions are made. It has the capacity to predict important clinical parameters like recanalization success and clinical outcome with the potential to influence clinical decision making in the hyperacute setting. Additionally, a parameter like this would easily be implementable in AI-based systems integrating multiple imaging parameters that surely will become available in the future.

### Limitations

A well-defined subgroup of complete occlusions of the MCA was selected in the present study. This was necessary to obtain a homogeneous cohort. Direct contact of fresh flooding contrast agent on the thrombus is the precondition to prevent the problem of a stationary blood column, which can be observed in occlusions of the internal carotid artery or basilar system; however, it limits the potential of generalizability and requires further studies that investigate the occlusions of the other sites. The existing problems of possible underestimation due to timing limitations, hemodynamic restriction or pseudo-occlusion cannot be avoided in a single-phase CTA, that is a further critical point of the study [19, 30].

The measurement of relative thrombus attenuation is not sufficiently possible for thrombi of very high density (e.g., due to calcification). Generally, perviousness measures cannot identify an increase of density by contrast agent if the baseline thrombus density is excessively high due to calcifications. Concordant to the low incidence of these kind of thrombi, there was no thrombus with visible calcifications included, but it should be considered for further analyses to find a new way to deal with calcified thrombi.

Further limitations affect the selection of covariates to predict clinical outcome. Beside demographical and technical (CTA thickness) aspects, angiographic variables such as procedure time as well as recanalization success were included for correction. Other parameters that were not assessed in the present study could also have an influence such as collateralization status. Not only the anterograde blood filling through the thrombus may impact the course of the dependent brain tissue, but also the blood supply through collaterals. Combining perviousness measurements with collateralization status could be realized in further studies to predict outcome and support therapeutic decisions.

The sample size of the present study was modest due to the single-center study design. Results should be confirmed in more extensive and multicenter cohorts.

## Conclusion

With CTA-index, a new imaging marker is introduced as a simplified measurement method of thrombus perviousness. It is easy to assess in acute stroke admission imaging and has the potential to serve as a new, additional prognostic parameter for angiographic and clinical outcome. Arterial-phase CTA is a standard procedure for diagnosing acute LVO, and the study conception showed the applicability of CTA-index assessment by (neuro)radiologists in daily clinical practice. As the CTA-index was associated with patient outcome, it therefore might support therapeutic and rehabilitative decisions, especially when combined with other standard imaging and clinical parameters.

## Abbreviations

CT: Computed tomography
CTA: Computed tomography angiography
ICC: Intraclass coefficient
LVO: Large vessel occlusion
MCA: Middle cerebral artery
mRS: Modified Rankin scale
mTICI: Modified thrombolysis in cerebral infarction
NIHSS: National Institutes of Health Stroke Scale
